# COVID-19 and its ramifications for cancer patients in low-resource settings: Ghana as a case study

**DOI:** 10.3332/ecancer.2020.ed99

**Published:** 2020-04-20

**Authors:** Nuworza Kugbey, Naomi Ohene-Oti, Verna Vanderpuye

**Affiliations:** 1 School of Public Health, University of Health and Allied Sciences, Ho, Ghana; 2 National Centre for Radiotherapy and Nuclear Medicine, Korle-Bu Teaching Hospital, Accra, Ghana; a http://orcid.org/0000-0002-0413-0350; b http://orcid.org/0000-0002-1433-0364; c http://orcid.org/0000-0003-3656-6965

**Keywords:** COVID-19, cancer care, financial toxicity, access to care, mental health, Ghana

## Abstract

The impact of COVID-19 on healthcare in low- and middle-income countries (LMICs) is a major challenge requiring urgent measures. Cancer care in LMICs, including Ghana, is faced with inadequate numbers of skilled healthcare professionals and essential material resources which negatively impacts the quality of healthcare and wellbeing of patients. In the face of COVID-19, cancer patients are likely to be affected in three key ways: access to healthcare, increased financial toxicity and increased mental health burden as a consequence of strict measures being implemented to contain the virus in Ghana, including partial lockdowns and social distancing. Some cultural beliefs regarding COVID-19 and its influence on the health and wellbeing of cancer patients have also been discussed. Measures by the government to lessen the burden on citizens and health workers are highlighted with possible recommendations for improvement in cancer care in Ghana and other LMICs during this pandemic.

Ghana is a low middle-income country (LMIC) in West Africa with a population of 30 million. Cancer incidence in Ghana is increasing as in all LMICs. There are two cancer registries located in Accra and Kumasi. A prevalence of 42,746 cases of cancer were reported over a 5-year period with the incidence of 22,823 new cases in 2018 [1]. The most prevalent is prostate and breast cancer in males and females respectively. Specialized oncology services are mostly provided in three health facilities throughout the country located in urban centres (2 public and 1 private). Some regional and other tertiary institutions perform some surgery and systemic therapy especially for breast cancer. The skilled oncology workforce in Ghana is below average as per many other sub-Saharan African countries [2].

Out of pocket expenditure for treatment is high among cancer patients even in the presence of the national health insurance (NHI) [3]. The NHI for cancer patients is a work in progress. It currently covers breast and cervical cancer and it is expected to cover paediatric cancer.

The emergence of COVID-19 has untoward implications for cancer care, more so in low-resource settings like Ghana. Invariably there are delays in scheduling surgeries, diagnostic procedures, radiotherapy and systemic therapy. As majority present with advanced disease, these unintentional delays are likely to result in less than optimal outcomes in the near future. Moreover, with a reduction in earning capacity and travel restrictions, a good portion of patients will default on their treatment as many rely on extended family income to support treatments. Ghana reported its first 2 cases on March 12, 2020 [4]. As at April 12^th^ 2020, the official number of confirmed cases are 408 and 8 deaths [4]. Majority of these confirmed cases are in Ghana’s capital Accra (87.5%) and in Kumasi (8%) with 57% as a result of community transmission [4]. This has led the Ghanaian government to enact closure of its schools and borders (land, air and sea), partial lockdowns of the two major cities mostly hit by the virus, provision of personal protective equipment to frontline healthcare workers, surveillance systems including contact tracing with testing (37,000 PCR based test performed to date) and mandatory quarantine of high-risk individuals to contain the pandemic.

The restriction on movement to and from the two major cities in Ghana where the cancer hospitals are located is likely to negatively impact the health and wellbeing of cancer patients. Access to healthcare has been linked to the overall quality of life and survival of cancer patients [5].

The economic fallout from this pandemic is likely to worsen the financial toxicity reported among cancer patients across the world [6]. As the prices of goods and services have increased due to the lockdown, the government of Ghana has introduced free water, subsidized electricity, tax-free reliefs to cushion health workers, small and medium scale businesses, bonuses for frontline health workers, preserving the food chain supply, free meals for the vulnerable with the aid of civil society organizations (CSOs) from April to June, 2020 [7]. This generous support may not be available to many LMICs faced with similar challenges. Hopefully this will lessen the impact of financial loss to our cancer patients who require care during this period and in the immediate post pandemic period.

The difficulties in accessing oncology care, economic burden posed by the pandemic and many other factors associated with the pandemic and its consequences will likely result in significant psychological distress in cancer patients in the medium to long run. Within the few days of the partial lockdown in Ghana, patients receiving treatment at the specialized cancer hospitals were anxiously complaining to the media about reduced access to cancer facilities. Oncology centres worldwide have a difficult task of triaging patients based on risk stratification for radiotherapy, systemic therapy and routine follow-up visits during these difficult times, in an attempt to protect vulnerable patients and staff [8].

In Ghana, the situation is actually worse as we have limited skilled staff who run shifts to avoid a total collapse of services in case there is exposure to a COVID-19 patient or staff member. More so, cancer patients in Ghana are oblivious of the dangers of the increased risk of COVID-19 transmission and poorer outcomes in cancer patients and survivors [9]. For them, nothing could be worse than a cancer diagnosis, therefore delaying treatment seems like a double jeopardy. It is not unusual for a patient to present to the oncology department with symptoms suggestive of COVID-19 and needing to be triaged for testing, which unfortunately could take 3 to 5 days to process in either of the two designated testing centres in Kumasi and Accra. All of this places undue burden on the cancer workforce who currently do not have access to SARS-CoV-2 testing or sufficient PPE, as oncology departments are not considered frontline workers. The challenge is the majority of these patients have already delayed presenting for diagnosis as well as delayed initiating interventions [10]. With the current ban on imports, it is unnerving to think about the high possibility of drug stock being depleted as almost all our systemic therapy medications are imported. The fears and uncertainties about further delays in treatments could affect the mental health and quality of life of both patient and oncologists. Moreover, the ban on social gatherings including religious activities is likely to exacerbate their mental health state as socializing and spirituality play a significant role in the health and wellbeing of cancer patients in Ghana [11].

Traditional beliefs such as the consumption of large quantities of alcohol and strong faith to ward off the virus may worsen the plight of cancer patients if social distancing and personal hygiene are ignored. Also, of concern is the negative effects of alcohol on cancer outcomes. Other harmful beliefs including the suspected use of COVID-19 test samples for ritual purposes may further jeopardize containment efforts as people are refusing their samples to be taken. This is similar to beliefs among Nigerians leading to the boycott of polio vaccination in the early 2000s [12, 13]. We expect that some cancer patients will resort to unorthodox health practices especially outside of the locked down areas, further worsening their plight. In a country with lower than average health literacy rate, where spirituality and communal living abounds, our governments need to enact strict measures to protect the vulnerable.

## Conclusion

The COVID-19 pandemic is a wake-up call to decentralize some aspects of cancer care in Ghana and other LMICs and improve communication between patients and cancer care providers. It has brought to the fore the psychological needs of cancer patients and indeed healthcare professionals who have to make very difficult treatment decisions. This calls for the engagement of psychologists to deal with the psychosocial needs of cancer patients and their healthcare professionals. Multidisciplinary research using innovative ways to gather empirical data among cancer patients and health facilities to inform policy decision making regarding cancer in Ghana in the face of the pandemic and beyond is warranted.

## Funding statement

The authors declare that they have received no funding for this article.

## Conflicts of interest

The authors declare that they have no conflicts of interest regarding this work.
